# The potential influence of Atlantic salmon *Salmo salar* and brown trout *Salmo trutta* on density and breeding of the white‐throated dipper *Cinclus cinclus*

**DOI:** 10.1002/ece3.3958

**Published:** 2018-03-26

**Authors:** Anna L. K. Nilsson, Jan Henning L'Abée‐Lund, L. Asbjørn Vøllestad, Kurt Jerstad, Bjørn Mejdell Larsen, Ole Wiggo Røstad, Svein Jakob Saltveit, Thomas Skaugen, Nils C. Stenseth, Bjørn Walseng

**Affiliations:** ^1^ Department of Biosciences Centre for Ecological and Evolutionary Synthesis (CEES) University of Oslo Oslo Norway; ^2^ Norwegian Water Resource and Energy Directorate Oslo Norway; ^3^ Jerstad Viltforvaltning Mandal Norway; ^4^ Norwegian Institute for Nature Research Trondheim Norway; ^5^ Faculty of Environmental Sciences and Natural Resource Management Norwegian University of Life Sciences Ås Norway; ^6^ Freshwater and Inland Fisheries Laboratory Natural History Museum University of Oslo Oslo Norway; ^7^ Norwegian Institute for Nature Research Oslo Norway

**Keywords:** birds, breeding, predators, river, salmonids

## Abstract

Interactions between birds and fish are often overlooked in aquatic ecosystems. We studied the influence of Atlantic salmon and brown trout on the breeding population size and reproductive output of the white‐throated dipper in a Norwegian river. Acidic precipitation led to the extinction of salmon, but salmon recolonized after liming was initiated in 1991. We compared the dipper population size and reproductive output before (1978–1992) and after (1993–2014) salmon recolonization. Despite a rapid and substantial increase in juvenile salmon, the breeding dipper population size and reproductive output were not influenced by juvenile salmon, trout, or total salmonid density. This might be due to different feeding strategies in salmonids and dippers, where salmonids are mainly feeding on drift, while the dipper is a benthic feeder. The correlation between the size of the dipper population upstream and downstream of a salmonid migratory barrier was similar before and after recolonization, indicating that the downstream territories were not less attractive after the recolonization of salmon. Upstream dipper breeding success rates declined before the recolonization event and increased after, indicating improved water quality due to liming, and increasing invertebrate prey abundances and biodiversity. Surprisingly, upstream the migratory barrier, juvenile trout had a weak positive effect on the dipper population size, indicating that dippers may prey upon small trout. It is possible that wider downstream reaches might have higher abundances of alternative food, rending juvenile trout unimportant as prey. Abiotic factors such as winter temperatures and acidic precipitation with subsequent liming, potentially mediated by prey abundance, seem to play the most important role in the life history of the dipper.

## 1 INTRODUCTION

**Figure 1:**
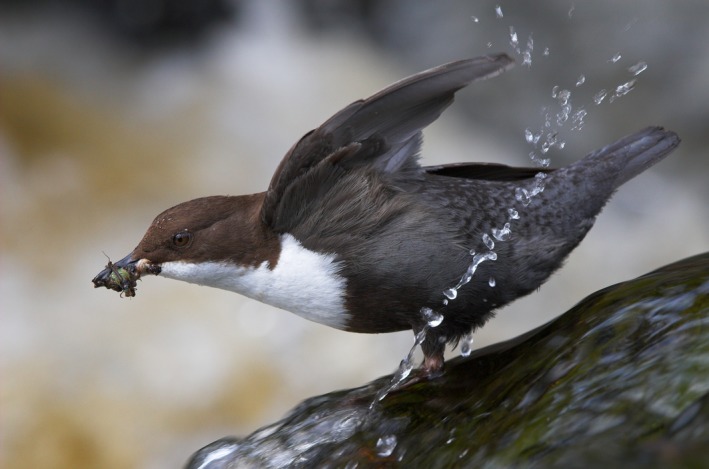
The white‐throated dipper *Cinclus cinclus*. Photo by Geir Rune Løvestad

In freshwater habitats, studies of predator and prey interactions are mainly focused on fish, amphibians, and invertebrates (Greenstreet & Tasker, 1996; Hildrew, 1992). Although often overlooked in aquatic predator–prey systems, many birds feed on fish as well as aquatic invertebrates. These are mainly birds belonging to the orders of Podicipediformes and Anseriformes (waterfowl; del Hoyo, Elliot, & Sargatal, 1992). Waterfowl actively compete with predatory fish for aquatic invertebrate prey and to some extent fish prey (Eadie & Keast, 1982; Kloskowski, 2011; LeBourdais, Ydenberg, & Esler, 2009; Strand, Chipps, Kahara, Higgins, & Vaa, 2008; Wagner & Hansson, 1998). In addition, the dippers (*Cinclus* sp, five species worldwide), belonging to the order Passeriformes, feed on aquatic invertebrates and fish (Øigarden, 2014; Tyler & Ormerod, 1994). American dippers *C. mexicanus* in Alaska enjoy increased reproductive performance on river reaches with spawning Pacific salmon (*Onchorhynchus* sp), due to birds foraging on eggs and juvenile salmon (Obermeyer, Hodgson, & Willson, 1999; Obermeyer, White, & Willson, 2006). The proportion of fish in the diet of the white‐throated dipper *C. cinclus* (Figure 1; hereafter dipper) is, however, relatively small (Tyler & Ormerod, 1994), but might due to its larger body size contribute substantially to the overall prey intake. The dipper mainly forages in the same habitat as juvenile Atlantic salmon *Salmo salar* (hereafter salmon) and brown trout *Salmo trutta* (hereafter trout; Heggenes, Saltveit, Bird, & Grew, 2002), all of whom prey on aquatic invertebrates. Indeed, the most important prey groups, mayfly (Ephemeroptera), and stonefly nymphs (Plecoptera) and caddies fly larvae (Trichoptera), are shared prey by salmonids and dippers (Aas, Klemetsen, Einum, & Skurdal, 2011; Klemetsen et al., 2003; Ormerod, Efteland, & Gabrielsen, 1987). In this study, we focus on the potential for interspecific interactions in a system where the changed abiotic conditions might have disturbed the established ecological balance.

Acidic precipitation has severely affected freshwater resources in southern Scandinavia since the 1920s (Dahl, 1920; Muniz, 1990; Nybø, Staurnes, & Jerstad, 1997; Wright, 1983). As a consequence, many rivers lost or partly lost biota that was not resistant to acidification (Muniz, 1990). Salmon were locally extinct in 40–50 rivers and streams in southern Norway in the 1970s (Hesthagen, Larsen, & Fiske, 2011; Muniz, 1984). Initiatives in the early 1980s with liming of lakes and rivers, in addition to reduced atmospheric deposition (Garmo, Skancke, & Høgåsen, 2013), and subsequent restocking of salmon, led to recolonization of salmon in rivers where it formerly was extinct or much reduced (Hesthagen et al., 2011). Over time, the population size of salmon has increased in these rivers. Trout is less affected by acidification than salmon (Poléo et al., 1997), and viable populations were retained in many locations where salmon became extinct (Hesthagen et al., 2011; Larsen, Hesthagen, Thorstad, & Diserud, 2015). The dipper, along with many other bird species, underwent a period with thinning of eggshells and subsequent reduction in fitness (Muniz, 1990; Nybø et al., 1997; Øigarden & Linløkken, 2010; Ormerod, O'Halloran, Gribbin, & Tyler, 1991; Ormerod & Tyler, 1993) due to aluminum and heavy metal body accumulation (Nybø, Fjeld, Jerstad, & Nissen, 1996), possible scarcity of calcium‐rich prey (Ormerod et al., 1991), and alterations in the macroinvertebrate prey community (Buckton, Brewin, Lewis, Stevens, & Ormerod, 1998; Muniz, 1990; Ormerod et al., 1991). Breeding on acidic streams leads to reduced clutch sizes, delayed breeding, and reduced nestling growth compared to on circumneutral streams (Ormerod et al., 1991). However, climate has so far been the most important factor explaining fluctuations in population size in Scandinavia (Nilsson et al., 2011; Saether et al., 2000).

The recovery of salmon populations after a period of extinction introduces the potential for renewed interactions with other vertebrates. One of these is the dipper, which in our study population was not as seriously affected by acidification as the salmonids. We hypothesize that the dipper might actually have been benefitted from relaxed interactive pressure from salmonids during the period when acidification lead to loss of the salmon population and a potential reduction in the trout population. Here, we investigate whether the abundance of juvenile salmonids influences the number of breeding dippers and their reproductive success in the River Lyngdalselva in southernmost Norway. The breeding population of dippers has been monitored annually since 1978, providing information on breeding attempts and breeding success of all dipper pairs in the system (Nilsson et al., 2011). In Lyngdalselva, the salmon were probably extinct or nearly extinct, but the population started to recover in 1993, 2 years after liming was initiated (Hesthagen et al., 2011). The trout population was probably not extinct as juvenile trout was observed when monitoring of the river started. We use long‐term monitoring data on the density of juvenile salmonids at fixed monitoring stations to test the effect of salmonids on the dipper. These data are available both below and above the migratory barrier (the waterfall Kvåsfossen) that restricts anadromous salmonids to the lower reaches of the river. Given this barrier, the river provides us with a comparative setup (a natural experiment) where we compare dipper success below the salmonid migratory barrier as well as in the “control” reaches upstream the barrier. Data from below the barrier are also used in a before and after salmon recolonization comparison. Experimental and control reaches are by necessity located at different tributaries and altitudes set by the migratory barrier and limit the natural river system as a comparison. However, the substantial gap in research on fish–bird interactions and the rare opportunity of unique and complete bird and fish data collected over a long time (1992–2014) in the same river system compensate for the limitations of “experimental” design.

## 2 METHODS

### 2.1 Study species

The distribution of salmon and trout overlaps in large parts of Europe (Jonsson & Jonsson, 2011; Klemetsen et al., 2003). Salmon is anadromous and spawns in freshwater during autumn (Aas et al., 2011). Trout can also be anadromous, but freshwater resident populations are most common (Klemetsen et al., 2003). Trout also spawns in autumn, approximately 2–3 weeks before salmon. The life history is very similar in salmon and trout and can be summarized as follows: Eggs are deposited in the river gravel during autumn. The eggs develop in the gravel during winter and hatch in spring. The hatched juveniles (yolk‐sac fry) live in the gravel for some time (weeks) until they become swim‐up fry and establish territories during early summer. These young individuals are referred to in the text as fry, but are often called young‐of‐the‐year, or 0 + parr. Larger juveniles are also called parr (or ≥1+), and this group of juveniles is in the text referred to as parr. Fry and parr have the potential to compete with the breeding dippers, being 3–15 cm in length (Jonsson, Jonsson, Brodtkorb, & Ingebrigtsen, 2001). Parr may metamorphose into smolt, which is a stage that is physiologically and morphologically preadapted to marine life. Following the smoltification, individuals migrate to sea during spring. The salmon returns to the native rivers after one or several winters at sea (Klemetsen et al., 2003; Thorstad, Whoriskey, Rikardsen, & Aarestrup, 2011). The trout has a more variable marine life and may return to freshwater after some months or several years in coastal environments (Thorstad et al., 2016). Both salmon and trout are iteroparous (can spawn several times during their lifetime). After spawning, the fish return to sea, either immediately or early next spring. The trout is a partial migrant, with some individuals (both males and females) completing the life cycle without migrating to the sea, whereas many make repeated migrations between the river and the sea during their life (Klemetsen et al., 2003). Some salmon males also remain stationary in freshwater. Both salmon and trout are harvested in a recreational fishery that is strongly regulated. Above the waterfall that functions as a barrier to migration in Lyngdalselva, there is a population of freshwater resident trout.

The juvenile stages of salmon and trout use a wide range of habitats in rivers and streams, but the smaller fry are more restricted to areas with low water velocity (<0.1 m/s) and shallow depths (<20 cm) than the larger parr (Finstad, Armstrong, & Nislow, 2011; Heggenes et al., 2002). Salmon and trout parr are territorial, and the availability of suitable territories will therefore limit the number of parr that a river can support (Elliott, 1994; Jonsson & Jonsson, 2011). This leads to strong density dependence that determines the carrying capacity of the river. There is also potentially strong competition between salmon and trout (Armstrong, Kemp, Kennedy, Ladle, & Milner, 2003).

The dipper is a common passerine bird in mountainous regions across the Palearctic. Its size is in the range of 50–70 g. Breeding is restricted to the immediate proximity of fast‐flowing rapids in early spring. Dippers build a nest with an outer part and an inner part. The outer part can be used for several years, whereas the inner is rebuilt each year. The female lays up to 7 eggs, most commonly 5 eggs and incubate them after the clutch is fully laid. The eggs hatch after 17 days, and the nestlings remain in the nest for another 20 days until fledgling (Tyler & Ormerod, 1994). Both parents feed the offspring, apart from in instances of polygyny where the female is providing most of the parental care. The dipper is dependent on open water for foraging and is therefore sensitive to temperatures and ice cover. Part of the population undertakes short migratory movements in autumn, while others remain on or close by the breeding grounds. Migrants from the study population have been recovered in southernmost Sweden, Denmark, Poland, and northern Germany.

### 2.2 Study population and data

**Figure 2:**
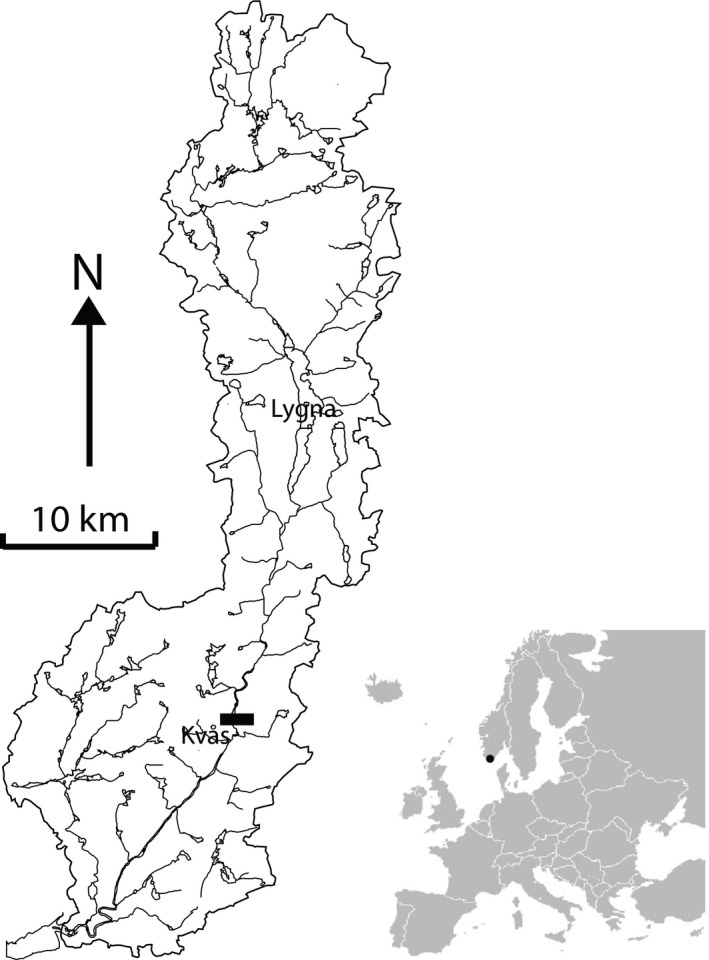
Map over the Lygna watershed. The salmonid migratory barrier at Kvås waterfall is marked in black

The study was conducted in the River Lyngdalselva in southernmost Norway (58°08′–58°40′N, 6°56′–7°20′E; Figure 2). Trout inhabits the whole river system, whereas the river is carrying migratory salmon and trout up to the waterfall Kvåsfossen approximately 20 km from the river mouth. The other recorded fish species in River Lyngdalselva are European eel *Anguilla anguilla*, ninespine stickleback *Pungitius pungitius*, threespine stickleback *Gasterosteus aculeatus,* and an unknown lamprey species. Observations from the tributaries Møska, Litlåna, and Skurvåna were excluded due to low numbers of dippers and fish observations of varying quality.

Annually, each autumn since 1991, juvenile salmon and trout have been sampled at fixed stations both below and above the migration barrier in the river by wading upstream using a backpack electrofishing unit delivering pulse of 1,200 V at a frequency of 86 Hz. The stations varied in size, between 124–428 m^2^ (mean area; Appendix S1). Based on length frequency histograms, the catch of salmon and trout was separated into two groups, fry and parr. The density (number per 100 m^2^) for each group of juvenile fish was estimated by the successive removal method averaged for the areas below or above the migratory barrier at Kvåsfossen waterfall (three removals, Appendix S2; Zippin, 1958; Bohlin, Hamrin, Heggberget, Rasmussen, & Saltveit, 1989).

The breeding population of dippers has been studied since the early 1970s, and the monitoring has been standardized since 1978 (see Nilsson et al., 2011 for more information). The study population ranges from the river outlet in Lyngdalsfjorden at the coast to 60 km inland and 700 m above sea level. The population size has fluctuated between 18 and 101 breeding pairs (defined as the number of breeding females, as males might occasionally be polygynous) during the study period 1978–2014. Almost all (94%) breeding birds are captured in mist nets at first sighting and ringed with a metal ring and an individual color code, enabling individual recognition at later encounters without having to catch the bird again. Within the river system, the breeding outcome of almost all occupied nests is known and nearly all nestlings are ringed. A breeding attempt is defined as positive when the inner nest is completed with a lining of leaves and as successful if it produces chicks and failed if it does not.

Dippers are restricted to nest sites in the vicinity of fast‐flowing water. There are therefore a limited number of potential territories with suitable nest sites, namely 120 in the River Lyngdalselva. The migratory salmonid‐carrying part contains 17 of these breeding territories. This includes 12 territories with direct access to the salmon‐carrying river as well as five territories with indirect access, where the territory is not located immediately on the salmon‐carrying river, but as the neighboring territory upstream in a tributary. Access to the salmon‐carrying main river is likely important for foraging success.

To investigate the effect of climate, we used mean annual winter temperature, defined as the average temperature during December–February. Meteorological data during 1977–2014 was recorded by the observation stations at Konsmo‐Eikeland (58°15′N, 7°19′E; 1978–1989), Konsmo‐Hægeland (58°16′N, 7°18′E; 1990), and Konsmo‐Høyland (58°16′N, 7°22′E; 1993–2014) located in the immediate proximity of the study area and was provided by the Norwegian Meteorological Institute (http://om.yr.no/verdata/).

This work has been carried out in accordance with Norwegian law and legal requirements, including those relating to conservation and animal welfare.

### 2.3 Statistics

For the sake of simplicity, the dipper population in the salmon‐carrying part of the river is hereafter shortened as “downstream dipper population” and in the nonsalmon‐carrying part “upstream dipper population”. Presented means are accompanied by standard deviations. All statistics were run in the program R, version 3.2.2 (R Core Team 2015), with add‐on packages “glmmADMB” for generalized linear mixed models (Bolker, Skaug, Magnusson, & Nielsen, 2013) and “MuMIn” for model averaging (Barton, 2016).

First, we explored the salmonid and dipper data for temporal trends with least‐squares regression analysis. Thereafter, we tested for correlations between the estimated average density of the fry and parr in the downstream as well as upstream sections of the river, and (1) the dipper population size, and (2) the reproductive output the next spring, respectively.

We fitted generalized linear models with Poisson error distributions, to explain (1) the variation in the size of the downstream dipper population with the annual density of salmonid fry and parr, respectively. Furthermore, we used a similar approach to explain (2) the variation in the size of the upstream dipper population with the annual density of trout fry and parr. Because the dipper population is highly dependent on fluctuations in winter temperature (Nilsson et al., 2011), we also included the mean annual winter temperature and the interaction between salmonid density and winter temperature among the predictors. In addition, we used model averaging and Relative variable Importance, RI, to evaluate the importance of the predictors. The number of variables, including interactions with winter temperature, was higher than in the other analyses, and we therefore only report the results from the model averaging process.

To investigate the ratio of failed versus successful breeding attempts, we used generalized linear models with binomial error distributions. In order to address (1) the temporal trend in reproductive output before and after salmon recolonized the river, and (2) the association between reproductive output and the density of salmonid fry and parr, we fitted generalized linear mixed models with Poisson and negative binomial error distributions, respectively. To account for the variation in the divergent occupancy rates of the 120 territories, territory was used as random effect. In the larger dataset addressing (1), we also accounted for the presence of individuals breeding more than once in the study system. Therefore, territory and individual were used as crossed random effects in the models.

## 3 RESULTS

The median downstream dipper population size was 10 breeding pairs (mean = 8.6 ± 3.9), and it fluctuated between 3 and 16 breeding pairs. In the upstream dipper population, the median number of breeding pairs was 49 (mean 53.4 ± 24.1) and it fluctuated between 19 and 98 breeding pairs. The density of salmon fry had an average of 27.3 fish per 100 m^2^ river stretch (*SD* = 23.3), while the average of salmon parr was 7.2 fish (*SD* = 7.9; Appendix S2). For downstream trout fry and parr, the average density was 23.0 (*SD* = 14.1) and 4.3 fish (*SD* = 2.3) per 100 m^2^ (Appendix S2), respectively, while the upstream trout fry and parr had an average density of 22.4 (*SD* = 17.2) and 6.1 fish (*SD* = 4.9; Appendix S2), respectively.

### 3.1 Impacts on dipper population size

**Figure 3:**
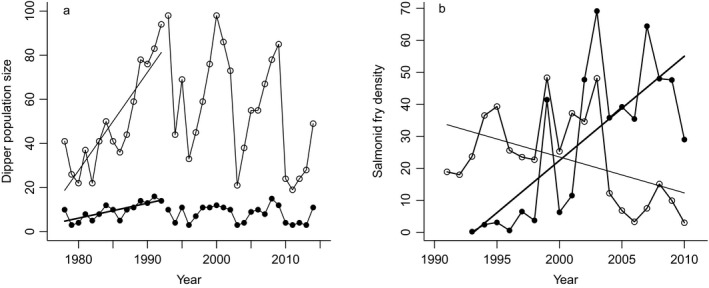
The time series of (a) the dipper breeding population downstream (filled symbols) and upstream (open symbols) the salmonid migratory barrier 1978–2014, (b) the annual density per 100 m^2^ of salmon (filled symbols, thick lines) and trout fry (open symbols, thin lines), downstream the migration barrier 1993–2010 in River Lyngdalselva. Significant trends are denoted with solid lines

There was no temporal trend in the total size of the downstream and upstream dipper populations over the dipper study period 1978–2014 (Figure 3a; downstream: *df* = 35, *t* = −0.5, *p* = .6; upstream: *df* = 35, *t* = 0.9, *p* = .4). The densities of salmon fry and parr increased over the study period (fry: *b* = 3.3, *df* = 16, *t* = 4.5, *p* = .0004, Figure 3b; parr: *b* = 1.2, *df* = 17, *t* = 6.9, *p* < .0001). There was a decrease in downstream trout fry density (Figure 3b; *b* = −1.1, *df* = 18, *t* = −2.3, *p* = .04), but not in downstream trout parr density (*df* = 18, *t* = −1.5, *p* = .15). Upstream, there was a decrease in trout parr (*b* = −0.4, *df* = 18, *t* = 2.4, *p* = .026), while there was no density change in trout fry (*df* = 18, *t* = 0.5, *p* = .6).

The size of the dipper populations downstream and upstream the salmonid migratory barrier was strongly correlated during the whole study period (*df* = 35, *r* = .83, *p* < .0001). As salmon recolonized the river from 1993, we reanalyzed the data for the periods 1978–1992 and 1993–2014 separately. The correlation between dippers downstream and upstream in both periods was almost equally strong (1978–1992: *df* = 13, *r* = .91, *p* < .0001; 1993–2014: *df* = 20, *r* = .86, *p* < .0001). Both dipper populations increased in size during 1978–1992 (Figure 3a; downstream: *b* = 0.7, *R* ^2^ = .58, *df* = 13, *t* = 4.3, *p* = .0009; upstream: *b* = 4.5, *R* ^2^ = .76, *df* = 13, *t* = 6.4, *p* < .0001), but there was no temporal trend after salmon recolonized downstream (Figure 3a; *b* = −0.08, *R* ^2^ = .02, *df* = 20, *t* = −0.6, *p* = .5), or upstream 1993–2014 (Figure 3a; *b* = −1.5, *R* ^2^ = .15, *df* = 20, *t* = −1.9, *p* = .07).

**Figure 4:**
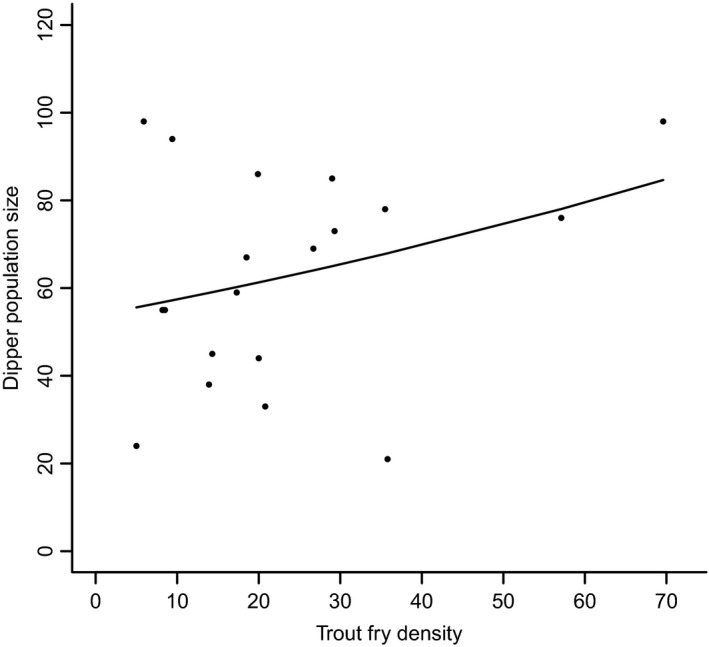
The association between the dipper population size and the annual density per 100 m^2^ of trout fry upstream the salmonid migratory barrier, 1991–2010 in River Lyngdalselva

The downstream dipper population size was not influenced by the density of juvenile fish; instead, it was influenced by the mean winter temperature (mean winter temperature: *b* = 0.2, *z* = 3.6, *p* = .0004; model averaging ΔAIC < 4; mean winter temperature RI = 1.0, trout fry RI = 0.21, salmon parr RI = 0.20, salmon fry RI = 0.11, trout fry × mean winter temperature RI = 0.10, trout parr RI = 0.09, salmon parr × mean winter temperature RI = 0.07). However, the upstream dipper population was positively affected by the mean winter temperature and positively affected by the density of trout fry (Figure 4; trout fry: *b* = 0.008, *z* = 4.6, *p* < .0001; mean winter temperature: *b* = 0.21, *z* = 6.1, *p* < .0001). The importance of trout fry was supported by model averaging (ΔAIC < 4; mean winter temperature RI = 1.0, trout fry RI = 1.0, trout parr RI = 0.34, trout fry × mean winter temperature RI = 0.12, trout parr × mean winter temperature RI = 0.09). Note that the relationship between the size of the upstream dipper population and the density of trout fry is dependent on the years 1999 and 2000 (Figure 4; 1999: hat = 0.27, 2000: hat = 0.51; see also Appendix S3).

### 3.2 Impact on dipper reproductive output

**Figure 5:**
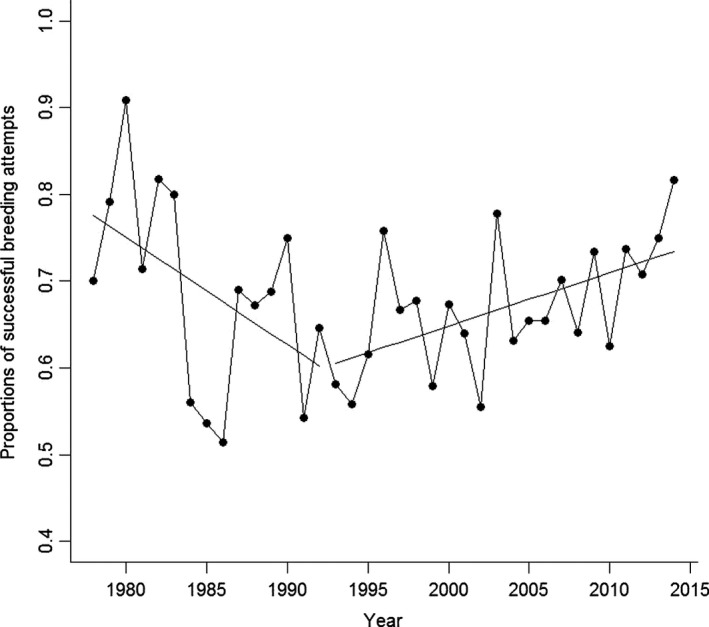
The rates of successful versus failed breeding attempts for the upstream dipper population, where significant trends are denoted with solid lines, 1978–1992 and 1993–2014 in River Lyngdalselva

In spite of the slight decline in the number of successful dipper breeding attempts in the downstream population after 1993, there was no difference in success rate between 1978–1992 and after 1993 when salmon recolonized this river section (0.85 and 0.80, respectively; Chi‐sq = 0.67, *df* = 1, *p* = .4). There was a difference in the number of successful breeding attempts upstream between 1978–1992 and after 1993 (0.70 and 0.64, respectively, Chi‐sq = 5.3, *df* = 1, *p* = .02). Using logistic regression, we tested whether there was a temporal trend in the rates of failed versus successful breeding attempts, 1978–1992 and after 1993. In the downstream population, there was no change in success rates during 1978–1992 (*b* = −0.05, *df* = 14, *z* = −0.8, *p* = .4) or after 1993 (*b* = 0.03, *df* = 21, *z* = 0.8, *p* = .4). In the upstream population, there was a significant decline in success rates during 1978–1992 (Figure 5; *b* = −0.04, *df* = 14, *z* = −2.1, *p* = .03) and a significant increase after 1993 (Figure 5; *b* = 0.03, *df* = 21, *z* = 3.0, *p* = .003). The downstream dipper population overall had a higher success rate than the upstream population (downstream mean=0.78 ± 0.10; upstream mean = 0.68 ± 0.09; *t* = 4.0, *df* = 52, *p* = .0002).

There was no difference in reproductive output, measured as the number of chicks in successful broods downstream, before and after salmon recolonized the river in 1993 (before: 3.9 ± 1.4; after: 4.0 ± 1.3; *t* = −0.7, *df* = 248, *p* = .5). Neither was there any difference in the upstream dipper population (before: 3.9 ± 1.3; after: 3.9 ± 1.3; *t* = −0.9, *df* = 1013, *p* = .4). There was no temporal trend in reproductive output downstream during 1978–1992 (*z* = 0.8, *p* = .5, random effects: territory *SD* = 0.0004, individual *SD* = 0.0003) or after 1993 (*z* = 0.4, *p* = .7; random effects: territory *SD* = 0.09, individual *SD* = 0.0006). Upstream, there was also no temporal trend during 1978–1992 (*z* = 0.9, *p* = .4; random effects: territory *SD* = 0.0007, individual *SD* = 0.0003) or after 1993 (*z* = 0.2, *p* = .9; random effects: territory *SD* = 0.0004, individual *SD* = 0.0003).

There were also no significant associations between the dipper reproductive output (measured as brood size) and the density of juvenile salmonids in the downstream population (salmon parr: *z* = −0.03, *p* = 1.0; salmon fry: *z* = −0.04, *p* = 1.0; trout parr: *z* = −0.3, *p* = .8; trout fry: *z* = −0.4, *p* = .7). Using the total juvenile parr and fry density did not improve the association (parr: *z* = 0.09, *p* = .9; fry: *z* = −0.2, *p* = .8). Neither was there an association between juvenile trout and the upstream dipper population (trout parr: *z* = 0.2, *p* = .9; trout fry: *z* = −0.4, *p* = .7).

## 4 DISCUSSION

During the last decades, the number of breeding pairs and the reproductive success of dippers in the River Lyngdalselva have displayed strong annual fluctuations. At the same time, the density of juvenile salmon has increased. However, concomitant with the increase in juvenile salmon, there has been a decrease in juvenile trout in the river downstream the migratory barrier, probably due to negative competitive interactions with salmon (Armstrong et al., 2003; Hesthagen et al., 2017). However, overall there has been a significant increase in total density of juvenile salmonids in the river. This might potentially have led to increased competition between salmon parr and dippers for invertebrate food. Despite this large increase in salmonid recruitment since its recolonization in 1993, from nonexistent to large numbers, there was no effect on the population size of downstream breeding dippers, perhaps because salmonids to a larger degree feed on drifting prey. Neither did the density of juvenile trout nor the total juvenile salmonid density affect the number of breeding dippers downstream. Upstream, however, juvenile trout density was positively correlated with the dipper population size. Dippers therefore likely feed on juvenile trout, particularly on upper reaches where the habitat might be more marginal than in the lower reaches. It thus seems that in the wider downstream river reaches, where other food potentially is more abundant, predation on juvenile trout might not be as important. Juvenile trout is more agile than invertebrate prey, making them more difficult to catch. Dipper predation on fish, particularly trout, is well documented, particularly in the British literature (Ormerod, 1985; Ormerod & Tyler, 1991; Tyler & Ormerod, 1994). Excluding potential outlier years did not remove the ecological effect of trout on the size of the upstream dipper population (Appendix S3). The positive correlation between dipper and trout may also be driven by an unmeasured environmental driver. Such a driver may be related to climate or weather. The fluctuations in the size of the dipper population in Lyngdalselva are to a large degree the result of variation in winter weather. We are aware that the Poisson model in this paper is insufficient to fully describe the dipper population dynamics. A thorough discussion of the climatic impact on the population fluctuations in this population can be found in Gamelon et al. (2017) and Nilsson et al. (2011).

There were no indications that juvenile salmonid density (salmon, trout or total juvenile density) affected the dipper reproductive output in this study population, neither downstream nor upstream the migratory barrier. This is contrary to the situation in the closely related American dipper, where the presence of salmon had a positive effect on the breeding (Obermeyer et al., 2006). However, that study was conducted in Alaska, where semelparous Pacific salmon species are strongly influencing the freshwater and surrounding terrestrial ecosystems (Willson & Halupka, 1995). Interestingly, the effect of salmon seemed to be stronger on later stages of the American dipper breeding cycle, because nestlings on salmon reaches were heavier than nestlings on nonsalmon reaches while there was no difference in clutch size (Obermeyer et al., 2006). Also, Ormerod and Tyler (1991) observe that most fish were eaten by the (white‐throated) dipper during winter, which might explain why juvenile trout affects the size of the breeding population but not the reproductive output. Because of the complexity of our study system and the relative inaccessibility of the nest sites, it is not possible to measure the effect of salmonids on the nestling body condition in the present study. The positive effect of salmon on the dipper in Alaska is probably due to the large number of eggs deposited and their nutrient inputs from rotting carcasses of dead spawners, providing a large energetic subsidy to the local fauna.

In this study, we did not measure abundances of the dipper and salmonid prey source, the freshwater invertebrate fauna. The long‐term data this study is based on are collected for other purposes and not designed for studying the interactions between competitors or food sources. We would thus need long‐term invertebrate monitoring data. There is an established monitoring program for benthic fauna in Lyngdalselva, but the data are sporadic in the beginning of our time series (Schartau, Hindar, & Hellen, 2015; Walseng & Bongard, 2001). However, there are indications of increased invertebrate (*Ephemeroptera*,* Trichoptera* and *Plecoptera*) abundances and species composition after liming in conjunction with salmon recolonizing the river (unpublished data). Further, studies of obligate aquatic predators and benthic prey abundances have failed to show an effect of the predator on the prey in about half of the cases (Dahl & Greenberg, 1996). Salmon and trout are both drift and benthic feeders, while the dipper is a benthic feeder. When the number of fish is manipulated, for example trout, fish do not have a major impact on benthic abundances in running waters, which can be interpreted as salmonids do not impact the benthic community structure of macroinvertebrates in running waters (Allan, 1982a,b; Reice, 1982). Finally, the behavior and activity of some of the prey species are hypothesized to change in the presence of a predator, which leads to reduced availability for predators as well as researchers wanting to monitor prey (Dahl & Greenberg, 1996; Nystrom, Mcintosh, & Winterbourn, 2003).

The positive trend in the downstream dipper population before salmon recolonized the river was likely due to a series of harsh winters in the beginning of the study period followed by milder winters in the beginning of the 1990s (Nilsson et al., 2011), and probably not a result of absence of salmon. The trends in the upstream dipper populations before and in the upstream and downstream populations after the salmon recolonization event could very well be the result of acid precipitation and the liming program starting in 1991. Similarly, the upstream population displayed a decrease in breeding success rate 1978–1992 and an increase after liming was initiated. In the United Kingdom, acidification has been shown to lead to delayed breeding, smaller clutch size, and reduced nestling growth compared to breeding on circumneutral rivers (Ormerod et al., 1991). It is not possible to elucidate potential separate effects of salmon and liming on the dipper as they appear at the same time. However, the strong correlation between the numbers of dippers downstream and upstream throughout the study period indicates that the presence of migratory salmonids was not making occupation of territories downstream migratory barriers less attractive. Also, there are indications that some of the important prey groups have increased downstream and others upstream, positive signs of improved water quality (unpublished data).

The upstream dipper breeding success rate decreased before 1993 and increased after, but given the lack of trend in upstream trout parr, the trend was probably not an indication of any negative impact of competition with trout. The decrease in success rate 1978–1992 would possibly be an effect of the acid precipitation and the increase 1993–2014 of the subsequently initiated liming program in 1991. The invertebrate surveys in the river showed that the acid‐sensitive mayfly *Baetis rhodani* were gone from the system in 1978 but had recolonized large parts already in 1998, probably as a result of the liming (Walseng & Bongard, 2001). The higher success rate downstream compared to upstream might be an effect of the presence of more marginal territories upstream, in addition to a lasting effect of acid precipitation above lime dispensers.

In conclusion, despite a large dependence on the abiotic conditions, the dipper seems to be positively affected by the biotic interactions with resident trout.

## CONFLICT OF INTEREST

None declared.

## AUTHOR CONTRIBUTIONS

ALKN conceived the idea. KJ, BW, SJS, and BML conducted the fieldwork. ALKN analyzed the data and did the statistical modeling. ALKN, JHLL, and AV wrote the manuscript. OWR, TS, and NCS provided editorial advice.
