# Supplementary material for: The potential influence of Atlantic salmon Salmo salar and brown trout Salmo trutta on density and breeding of the white‐throated dipper Cinclus cinclus
Source: Ecol Evol. 2018 Mar 26;8(8):4065–73. doi: 10.1002/ece3.3958 (PMC5916291; doi:10.1002/ece3.3958)
Supplement: Supplementary file 1 [file ECE3-8-4065-s001.docx]

Appendix S1. Average station size for standard sampling of juvenile Atlantic salmon *Salmo salar* and trout *S. trutta*. The location of each station is indicated, if it is located above or below the migratory barrier at the Kvås waterfall for anadromous salmonids. All stations have been sampled 20 years.

| **Station** | **Below/above** | **Mean area (m^2^)** |
| --- | --- | --- |
| 1 | Below | 138 |
| 2 | Below | 165 |
| 3 | Below | 138 |
| 4 | Below | 167 |
| 5 | Below | 151 |
| 6 | Below | 162 |
| 7 | Below | 131 |
| 8 | Below | 132 |
| 9 | Below | 146 |
| 10 | Below | 146 |
| 21 | Above | 123 |
| 22 | Above | 151 |
| 23 | Above | 157 |

Appendix S2. Summary of the estimated annual densities (number per 100 m^2^) of juvenile Atlantic salmon *Salmo salar* and trout *S. trutta*, fry and parr, downstream and upstream the migratory barrier at the Kvås waterfall. Station denotes the number of sampling stations included in the calculated mean density.

**Downstream the migratory barrier at Kvås waterfall**

| **Year** | **Stations** | **Trout parr** | **Trout fry** | **Salmon parr** | **Salmon fry** |
| --- | --- | --- | --- | --- | --- |
| 1991 | 10 | 4.4 | 18.9 | 0.0 | 0.0 |
| 1992 | 10 | 4.2 | 18.0 | 0.1 | 0.0 |
| 1993 | 10 | 2.1 | 23.7 | 0.0 | 0.2 |
| 1994 | 10 | 7.2 | 36.5 | 0.7 | 2.4 |
| 1995 | 10 | 4.3 | 39.3 | 1.7 | 3.1 |
| 1996 | 10 | 5.0 | 25.6 | 0.4 | 0.6 |
| 1997 | 10 | 5.4 | 23.5 | 0.9 | 6.5 |
| 1998 | 10 | 4.4 | 22.7 | 1.2 | 3.7 |
| 1999 | 10 | 4.0 | 48.3 | 1.1 | 41.4 |
| 2000 | 10 | 9.8 | 25.3 | 6.3 | 6.3 |
| 2001 | 10 | 5.5 | 37.2 | 1.9 | 11.5 |
| 2002 | 10 | 6.0 | 34.6 | 5.4 | 47.7 |
| 2003 | 10 | 7.9 | 48.1 | 14.6 | 69.1 |
| 2004 | 10 | 4.3 | 12.2 | 10.8 | 35.8 |
| 2005 | 10 | 0.5 | 6.8 | 4.4 | 39.2 |
| 2006 | 10 | 0.8 | 3.3 | 17.0 | 35.4 |
| 2007 | 10 | 1.5 | 7.5 | 7.2 | 64.4 |
| 2008 | 10 | 2.5 | 15.1 | 17.1 | 48.0 |
| 2009 | 10 | 2.3 | 9.9 | 21.1 | 47.6 |
| 2010 | 10 | 4.0 | 3.0 | 24.2 | 29.0 |

**Upstream the migratory barrier at Kvås waterfall**

| Year | Stations | Trout parr | Trout fry |
| --- | --- | --- | --- |
| 1991 | 3 | 1.3 | 2.8 |
| 1992 | 3 | 1.5 | 9.4 |
| 1993 | 3 | 0.9 | 5.9 |
| 1994 | 3 | 3.4 | 20.0 |
| 1995 | 3 | 2.8 | 26.7 |
| 1996 | 3 | 5.3 | 20.8 |
| 1997 | 3 | 14.2 | 14.3 |
| 1998 | 3 | 8.5 | 17.3 |
| 1999 | 3 | 5.5 | 57.1 |
| 2000 | 3 | 5.0 | 69.6 |
| 2001 | 3 | 4.7 | 19.9 |
| 2002 | 3 | 3.6 | 29.3 |
| 2003 | 3 | 11.3 | 35.8 |
| 2004 | 3 | 7.7 | 13.9 |
| 2005 | 3 | 1.8 | 8.2 |
| 2006 | 3 | 1.3 | 8.5 |
| 2007 | 3 | 3.8 | 18.5 |
| 2008 | 3 | 13.3 | 35.5 |
| 2009 | 3 | 8.5 | 29.0 |
| 2010 | 3 | 18.1 | 5.0 |

Appendix S3. Excluding the years 1999 and 2000, the upstream dipper population was affected by the interactions between mean winter temperature and trout parr and fry, respectively (trout parr: b=0.02, z=1.7, P=0.09; trout fry: b=-0.01, z=-1.8, P=0.07; mean winter temperature: b=0.08, z=1.5, P=0.1; trout fry x mean winter temperature: b=0.01, z=3.8, P=0.0001, Fig. A3.1a; trout parr x mean winter temperature: b=-0.04, z=-4.7, P<0.0001, Fig. A3.1b).
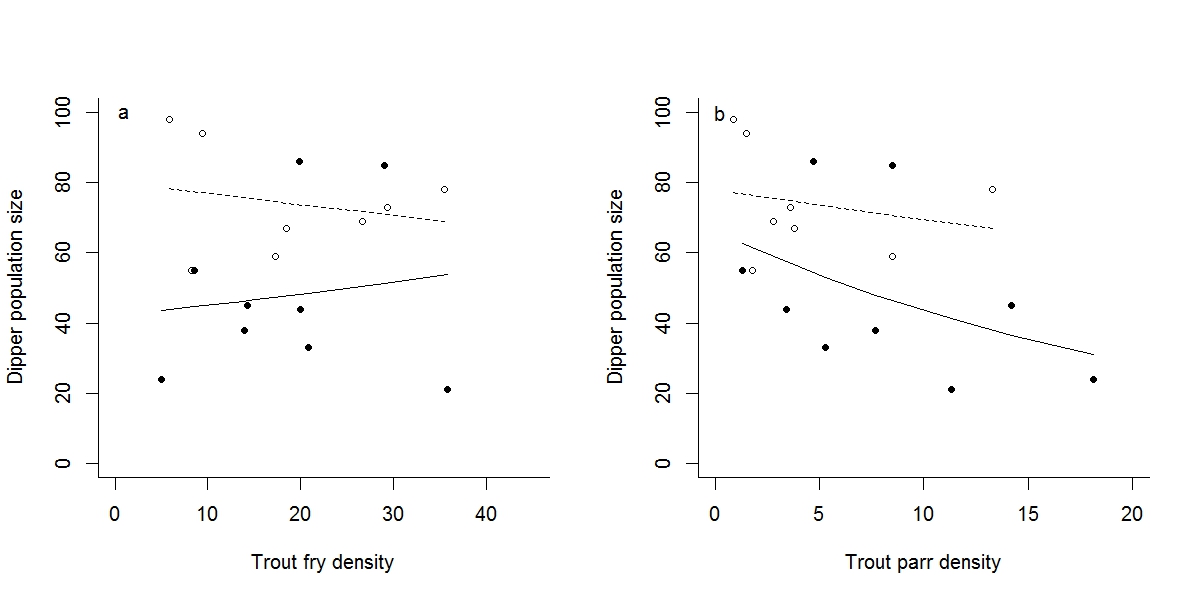


Fig. A3.1. An illustration of the effect on the size of the upstream dipper population of the interactions between mean winter temperature and the annual density of (a) fry and (b) parr, respectively, when the years 1999 and 2000 are excluded from the analyses. Filled symbols and solid lines depict mean winter temperatures below 0ᵒC and open symbols and broken lines temperatures above 0ᵒC.
